# Mr. Hodson's New Mode of Extracting a Stone

**Published:** 1802-05

**Authors:** T. Hodson

**Affiliations:** Lewes


### *To the Editors of the Medical and Phyfical Journal.*

GENTLEMEN,

IF you think the following defcription of an Inftrument, which I have contrived for the purpofe of extracting a Stone from the Bladder, worthy a place in your very ufeful Publication, I fhall be obliged to you to notice it the firft convenient opportunity.

It confifts of an inftrument made of iron, about 14 inches long, with a curve towards the point about 3 inches in length, fo as very much to refemble a common Sound, which I will, to enable me to defcribe with more brevity, call the Guide, and a piece of bladder, about 20 inches long, and 5 broad at the middle, but gradually becoming narrower, fo as at each end to be only 3 inches; this I will, for the fame reafon, call the Sling.

The

The ſling, or ſlip of bladder, being properly prepared by being ſoaked in warm water, and afterwards well oiled, one end of it is to be ſewed round the extremity of the guide, where there are two ſmall holes for that purpoſe, which after the inciſion has been made into the bladder in the uſual way, is to be introduced with the point turned towards the right iſchium; it is then to be carried forwards along the inferior part of the bladder till it has got beyond the ſtone, when the extremity of the guide to which the ſling is attached is to be raiſed and brought back over the ſtone, by which means the ſtone will be embraced by the ſling, and may, I conceive, be extracted. I need hardly obſerve, that I ſhould not propoſe to make uſe of this apparatus in preference to the forceps, excepting in caſes where the ſtone is found to be very large. In ſuch caſes, provided upon trial it ſhould be found practicable to extract a ſtone with it, two very great advantages would I think be attained by it; in the firſt place, the room which the forceps muſt neceſſarily occupy would be got rid of, and in the next the ſling, or piece of bladder in which the ſtone is enveloped, would afford conſiderable protection to the parts againſt the ſurface of the ſtone in its paſſage, which deſirable object would probably be promoted by means of a piece of tape fixed to each ſide of the ſling, about the middle, to prevent its folding back during the extraction.

The difficulty of meeting with an opportunity of making trial upon ſuch occaſions on the dead ſubject, in the country, will, I hope, plead my excuſe for offering this contrivance to the conſideration of your readers, without having firſt put it to the teſt. I am, &c.

*Lewes, March* 2, 1802. T. HODSON.

